# Post Bellum Problems

**Published:** 1918-10

**Authors:** 


### Post Bellum Problems.
### Physical, Mental and Moral Repair Work.

The raising of a large army from civilians has given us a good idea of the proportionately small but numerically enormous number of individuals who are, in one way or another defective. The training of those accepted, even provisionally has quite successfully repaired many of these defects, has shown the precautions as to branch of service or line of peace work within the ability of the partially disabled, as well as the hygienic and sociologic training and guarding also necessary. Not to mention tuberculosis, mainly in arrested or at least arrestable forms; hernias, piles, club feet, decayed teeth, adenoids, visual defects and the venereal diseases with which medical readers are familiar, the 1% or so of mental defectives who can be made fairly useful and kept fairly healthy and happy or who can become tramps,—and the lesson as to prostitutes is equally plain by inference and indirect military experience—are now a well understood problem for state care.

It is said that considerably more than half a million illiterates have been found and as the rudimentary English education of all soldiers has long been part of the military training, definitely provided for in Army Regulations, this defect will be well on the way to correction before the illiterate sails for France. In this connection, it may be said that a good deal of the trouble with pacifists of the lower grade has been found to be ignorance, often combined with actual illiteracy, though the ignorance is in regard to general information and inability to reason, rather than primary school studies. This type of pacifist is, as a rule, a well meaning, moral, religious, hard working boy but with very limited literal geographic and figurative intellectual horizons. What views they do hold, have been gained from teachers and other leaders a generation or two behind the times in conceptions of every kind, including the very practical present conception of the interrelationship of all nations of the globe. In a surprising number of instances, they scarcely realize that there is a war, or that it can by any possibility affect them or even the country. Having been limited to a ten-mile radius of movement, they think of 3500 miles as we do of interplanetary space. They cannot visualize or comprehend the man power of millions from an acquaintance of a few hundreds, nor the mechanic power and danger of modern military machinery from the feeble fire-arms, and simple hand-turned and horse-drawn implements with which they are familiar. The sophistries on which they have been brought up have never been measured against the straight edge of modern logic. A considerable number of these conscientious objectors need only a few weeks' association with well informed, keen minded youths to become aroused and to realize where they stand as American citizens

So much has been said as to the morals of our army, in a restricted sense, that we need not dwell on this phase of the subject. There is such a thing as compulsory morality just as there is compulsory education. It does not go very far, but it tides over a period at least. Morality in a broader sense depends largely on discipline and while most of us may dislike discipline and especially in military dosage, it has its advantages.

To a large degree, those subjected to the various forms of repair work, are able to pass along to others, after the war, either the general fact that it is necessary and can be done, or in some simple particulars, can actually put it into execution. But, for the most part, the work to be of permanent and general value, must be taken over by some branch of the government outside of the military establishment. In an advisory and, in many ways, a direct capacity, the medical profession must play an active part.

